# A chromosome-level genome assembly of *Amorphophallus konjac* provides insights into konjac glucomannan biosynthesis

**DOI:** 10.1016/j.csbj.2022.02.009

**Published:** 2022-02-15

**Authors:** Yong Gao, Yanan Zhang, Chen Feng, Honglong Chu, Chao Feng, Haibo Wang, Lifang Wu, Si Yin, Chao Liu, Huanhuan Chen, Zhumei Li, Zhengrong Zou, Lizhou Tang

**Affiliations:** a College of Biological Resource and Food Engineering, Center for Yunnan Plateau Biological Resources Protection and Utilization, Qujing Normal University, Qujing, Yunnan 655011, China; b College of Lifesciences, Jiangxi Normal University, Nanchang 330022, China; c Lushan Botanical Garden, Chinese Academy of Sciences, Jiujiang, China; d Key Laboratory of Plant Resources Conservation and Sustainable Utilization, South China Botanical Garden, Chinese Academy of Sciences, Guangzhou, China

**Keywords:** *Amorphophallus konjac*, Genome evolution, Whole-genome duplication, Glucomannan biosynthesis

## Abstract

*Amorphophallus konjac*, a perennial herb in the Araceae family, is a cash crop that can produce a large amount of konjac glucomannan. To explore mechanisms underlying such large genomes in the genus *Amorphophallus* as well as the gene regulation of glucomannan biosynthesis, we present a chromosome-level genome assembly of *A. konjac* with a total genome size of 5.60 Gb and a contig N50 of 1.20 Mb. Comparative genomic analysis reveals that *A. konjac* has undergone two whole-genome duplication (WGD) events in quick succession. Two recent bursts of transposable elements are identified in the *A. konjac* genome, which contribute greatly to the large genome size. Our transcriptomic analysis of the developmental corms characterizes key genes involved in the biosynthesis of glucomannan and related starches. High expression of cellulose synthase-like A, Cellulose synthase-like D, mannan-synthesis related 1, GDP-mannose pyrophosphorylase and phosphomannomutase fructokinase contributes to glucomannan synthesis during the corm expansion period while high expression of starch synthase, starch branching enzyme and phosphoglucomutase is responsible for starch synthesis in the late corm development stage. In conclusion, we generate a high-quality genome of *A. konjac* with different sequencing technologies. The expansion of transposable elements has caused the large genome of this species. And the identified key genes in the glucomannan biosynthesis provide valuable candidates for molecular breeding of this crop in the future.

## Graphical abstract

As a glucomannan-producing cash crop in many Asian countries, a chromosome-level genome assembly of *Amorphophallus konjac* is generated. Two whole-genome duplications in quick succession as well as recent bursts of transposable elements are discovered in the genome. Cellulose synthase-like A, Cellulose synthase-like D, mannan-synthesis related 1, GDP-mannose pyrophosphorylase and phosphomannomutase fructokinase are found to contribute to glucomannan synthesis.

**Figure:**
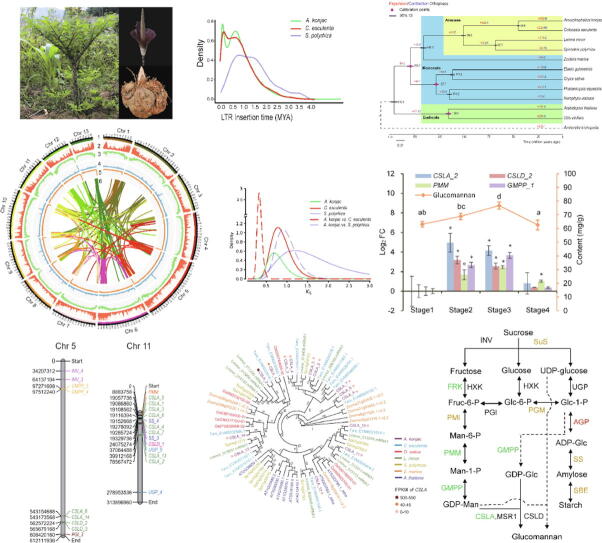

## 1 Introduction

**Fig. 1:**
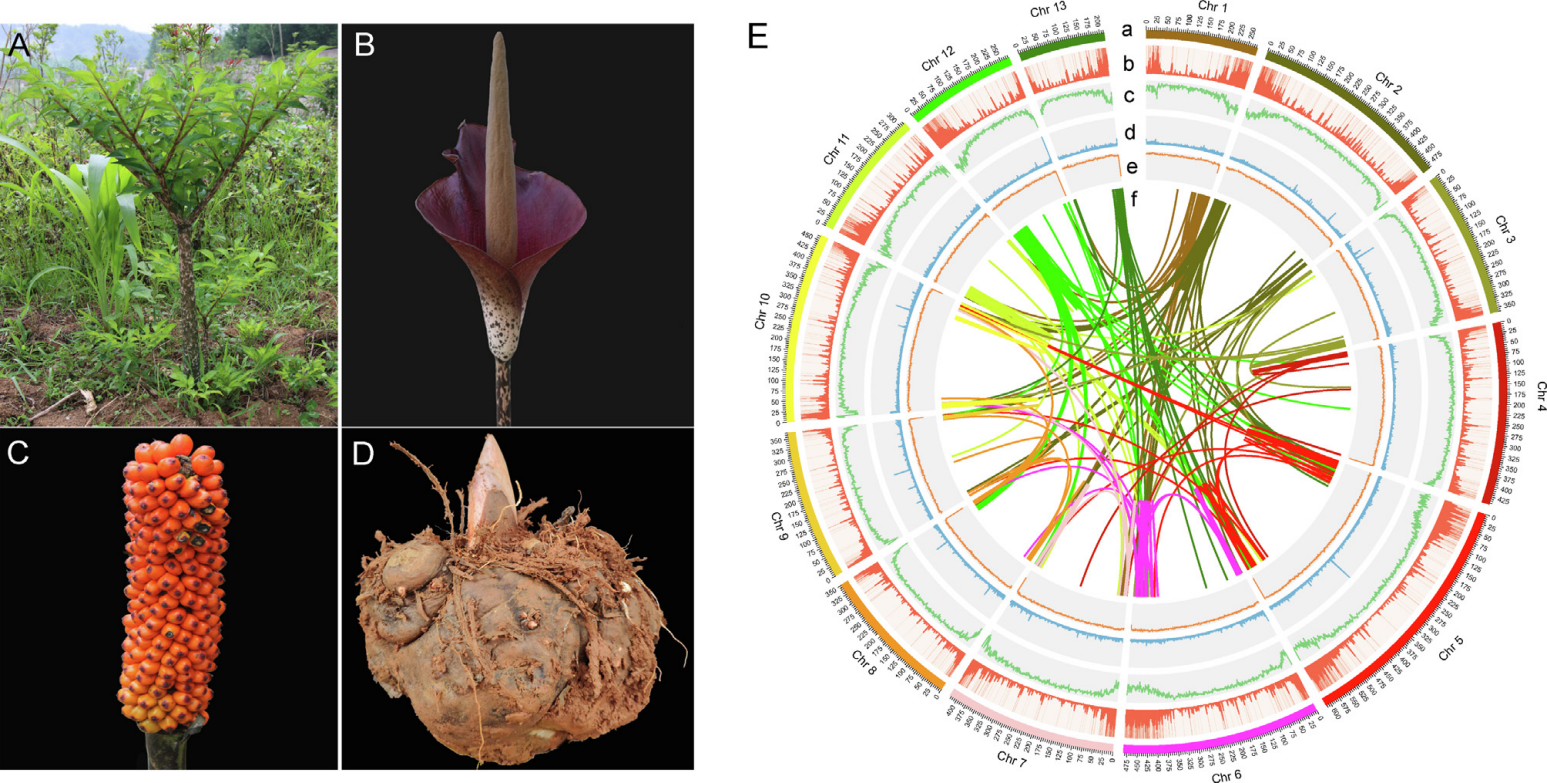
Leaf, flower, fruit and corm morphology, and the genome landscape of *A. konjac*. (A-D) The leaf, flower, fruit and corm morphology of *A. konjac*; (E) The genome landscape of *A. konjac*, (a) Length of each chromosome in megabases (Mb), (b) Gene density, (c) Repeat density, (d) Tandem repeat density, (e) GC content, (f) Intragenomic synteny information.

Carbon fixation by photosynthesis in green plants is fundamental for the ecosystem. Most plants store their photosynthetic product as starch, while species in genus *Amorphophallus* of the Araceae family are within the few plants that can accumulate large amounts of konjac glucomannan (KGM) [1]. This genus contains around 170 species characterized by a solitary leaf and an underground stem (corm) [2], [3]. For the large KGM content, the *Amorphophallus* corm has long been regarded as a non-calorie health food [1], [4]. In particular, *Amorphophallus konjac* (2n = 2x = 26) is the most important and widely utilized species of this genus [1]. The spathe of *A. konjac* is deep purple-red, and the oval-shaped fruit chamber turns from green to orange during ripening (Fig. 1A-1D). For its strong adaptability, the cultivation of *A. konjac* has expanded from China and Japan to Southeast Asia, including Thailand and Indonesia [5], [6].

KGM biosynthesis is a multistep process in which a series of enzymes convert substrates like sucrose into glucomannan. Previous analyses suggest that glucomannan is comprised of mannose and glucose with a ratio of 1.8:1, and with 11% of the mannosyl residues *O*-acetylated equally at position O-2 and O-3 [7]. Given its wide application as food and industrial materials, the KGM biosynthesis pathway and its regulation are of great interest. In *Arabidopsis*, glucomannan is a conserved cell wall mannan polysaccharide, and is synthesized by the cellulose synthase-like A (CSLA) family of enzymes [8], [9]. In addition, mannan-synthesis related 1 (MSR1) of *Arabidopsis* is supposed to be an optional cofactor for glucomannan synthesis, and the coexpression of *AtMSR1* and *AkCLSA3* in yeast significantly increase the amount of glucomannan relative to AkCLSA3 alone [10]. Using information from studies of *Arabidopsis*, the KGM biosynthesis pathway has been investigated based on enzyme analysis [11] and RNA sequencing [7], [12]. However, without a reference genome sequence for *A. konjac* and time-course RNA sequencing, the key genes involved in KGM biosynthesis and its regulation remain unclear.

Until now, there are up to 40 chloroplast genomes reported in the Araceae family, of which five chloroplast genomes of *Amorphophallus* species have also been sequenced [13], [14], [15], [16]. All these studies advance our understanding of genetic diversity, phylogeny and the genetic breeding of Araceae species [17]. However, the Araceae family which comprises a large number of diverse species with huge differences in genome sizes [18]. For example, *Spirodela polyrhiza* is an aquatic plant with a genome size of only 158 Mb, while species of other genera, such as *Colocasia* and *Amorphophallus*, have relatively larger genomes (2.40 Gb–15.48 Gb) [19], [20]. As the chloroplast only provides information on the unilateral inheritance, the genome data is more accurate in inferring evolutionary history of species. Genome expansion in plants is primarily driven by whole-genome duplication (WGD) events and the proliferation of transposable elements (TEs) [21]. The genomics architecture of *A. konjac* can provide additional insights into mechanisms underlying the genome expansion in genus *Amorphophallus* as well as the evolutionary history within Araceae.

To investigate mechanisms underlying such large genomes in genus *Amorphophallus* as well as the key gene involved in KGM accumulation, a high-quality chromosome-level genome of *A. konjac* was assembled. We annotated genomic characteristics of the assembly, and described the evolutionary history of the *A. konjac* genome. We also performed time-course transcriptomic analysis for the developing corms, and revealed the key genes associated with KGM biosynthesis and its regulation. One desirable characteristic of the *A. konjac* variety is little starch, but mostly glucomannan accumulated in the corms. The obtained results provide a basis for future molecular breeding to increase the glucomannan content via genetic engineering technologies, such as RNA interference and CRISPR-Cas*.*

## 2 Materials and methods

### 2.1 Genome sequencing

As Fuyuan county is one of the largest plantation areas of *Amorphophallus konjac* in China, we decided to choose the representative landrace in this region to perform the whole genome sequencing. One cultivated individual was collected from a plantation in Fuyuan county (25°35′36″ N, 104°5′32″ E), Yunnan province, China. Genomic DNA was isolated using a commercial DNA extraction kit (DP305; Tiangen, Beijing, China). The Illumina short-insert libraries were constructed with an insert size of 500 bp. For the PacBio sequencing, a 20 kb SMRT library was constructed and sequenced on a PacBio Sequel Ⅱ sequencer. In addition, the 10X Genomics sequencing libraries were produced and sequenced on the Ilumina HiSeq platform. At last, the High-through chromosome conformation capture (Hi-C) sequencing libraries were constructed and sequenced on a HiSeq 4000 sequencer.

### 2.2 *De novo* genome assembly

The genome size of *A. konjac* was estimated by the *K*-mer distribution analysis (*K* = 17) using 673 Gb of Illumina data. PacBio long reads were used to generate contig-level assembly by FALCON (https://github.com/PacificBiosciences/FALCON/). Illumina short reads were then used to polish the genome assembly with PILON v1.22 [22]. The Purge Haplotigs pipeline (https://bitbucket.org/mroachawri/purge_haplotigs/overview) was applied to remove redundant sequences that were formed due to the heterozygosity of genome sequences. To improve the continuity of the assembly, FragScaff (https://sourceforge.net/projects/fragscaff/files/) was used to construct scaffolds with the aid of sequences from the 10X Genomics libraries. Finally, the Hi-C sequencing data were used to cluster, orientate, and link the assembled sequences into 13 pseudo-chromosomes.

### 2.3 Genome quality assessment

To assess the assembly quality of the *A. konjac* genome, the coverage was calculated by mapping Illumina short reads to the assembly using Burrows-Wheeler Aligner (BWA) [23]. The completeness of the assembly was evaluated using Benchmarking Universal Single-Copy Orthologs (BUSCO) v10 [24]. The completeness of the genome assembly was also evaluated using the Conserved Core Eukaryotic Gene Mapping Approach (CEGMA) [25]. We calculated the long terminal repeat (LTR) assembly index (LAI) scores of genomes of *A. konjac, S. polyrhiza* and *Colocasia esculenta* using *LTR_retriever* [26].

### 2.4 Genome annotation

A combined strategy based on *de novo* search and homology alignment was used to identify the genome repeats. Tandem repeats were extracted using TRF v4.07b by *de novo* prediction [27]. A homology-based search for repeat sequences was further carried out using RepeatProteinMask and RepeatMasker v3.3.0 (www.repeatmasker.org/). LTR retrotransposons in the *A. konjac, S. polyrhiza* and *C. esculenta* genomes were initially identified using LTRharvest and LTR_FINDER. The non-redundant LTR-RTs were then generated, and the timing of their insertion was estimated using *LTR_retriever* [26].

*De novo*, homology based and RNA-seq assisted predictions were used to annotate protein-coding genes. For *de novo* identification, five gene prediction programs (i.e. Augustus, GlimmerHMM, SNAP, Geneid and Genscan) were used to predict gene models. Proteins of six sequenced plants, *Arabidopsis thaliana*, *Oryza sativa*, *Zostera muelleri*, *Zostera marina*, *Lemna minor* and *S. polyrhiza* were aligned to the assembly of *A. konjac* using tBlastN. For the RNA-seq based annotation, RNA-seq data was aligned to the assembly and gene models were generated using Cufflinks [28]. In addition, transcriptome reads were assembled, and ESTs were aligned against the assembly using PASA [29]. The non-redundant reference gene set was generated by merging genes predicted by three methods with EvidenceModeler v1.1.1 [29]. Potential functions of the genes were annotated with the non-redundant protein database (Nr), KEGG, Swissprot, Interprot and Pfam databases.

Several methods were applied to identify the noncoding RNAs in the *A. konjac* genome. The tRNAs were predicted using the program tRNAscan-SE, and snRNA and rRNA genes were identified by searching against the Rfam database using the infernal software [30]. The microRNA genes were annotated using BLASTN based on the datasets of miRBase (www.mirbase.org).

### 2.5 Analysis of gene families and phylogenetic evolution

To investigate the evolutionary position of *A. konjac*, we downloaded the genome sequences of 11 plants. Orthologous genes of *A. konjac* and other plants were identified using OrthoFinder v2.2.7 [31]. A maximum-likelihood (ML) phylogenetic tree was constructed using IQ-TREE v1.6.11 [32]. Divergence time between species was estimated by BEAST v2.6.0 [33], and time calibrations were determined using the TimeTree database (http://www.timetree.org/). The BEAST analysis was run for 100 million generations and sampled every 10,000 generations. Gene family expansions and contractions were calculated using CAFE [34].

### 2.6 Analysis of whole genome duplication events

For inferring the WGD events in *A. konjac* genome, wgd software was used to construct a distribution of *K*s values [35]. The curves of *K*s distribution were fitted with Gaussian mixture models. To assess the collinearity among *A. konjac, S. polyrhiza* and *C. esculenta*, syntenic blocks among the three species were identified using MCScan [36].

### 2.7 RNA-seq and gene expression analysis

To determine the key genes in KGM biosynthesis, time-course RNA-seq was performed for developmental corms collected from four stages of the vegetative growth circle: dormancy stage (stage 1), ‘changing head’ stage (stage 2), corm expansion stage (stage 3) and maturity stage (stage 4). And three or four individuals were sampled at each stage as biological replicates. The total RNA was extracted with the RNAprep Pure Plant Plus Kit (Tiangen), and 1 μg of RNA for each sample was prepared to construct the RNA-seq libraries using a NEBNext Ultra™ RNA Library Prep Kit, after which PE150 sequencing was conducted on the Illumina HiSeq 4000 platform. For quality control, low-quality bases, adapter duplications, and potential contaminants were removed. The remaining clean reads were then mapped onto the reference genome. The gene expression level was quantified as FPKM using featureCounts v1.5.0 [37]. Differential expression analysis and PCA were conducted using the R package DESeq2 v1.16.1 [38]. Heatmap and GO enrichment analysis were generated by TBtools v1.082 and clusterProfiler v3.4.4, respectively [39], [40].

### 2.8 Determination of the glucomannan content and RT-qPCR

The extraction of glucomannan was adopted from the previous report [7]. Briefly, frozen corm samples were ground to a fine powder using a ball mill. Then 0.2 mol/L sodium carbonate solution was added to facilitate the formation of KGM hydrogels. After heating at 80 ℃ for 3 to 5 min, vacuum filtration was repeated to isolates the dissolved KGM hydrogels. The KGM hydrogels were washed in 95% ethanol and subsequently filtered using filter papers. Glucomannan was extracted from the filtered alcohol insoluble residue and dried by hot air. The glucomannan content was measured by UV spectrophotometry at 570 nm using commercially available KGM as reference standards.

For RT-qPCR, RNA was extracted, and cDNA was obtained by reverse transcription using the PrimeScript™ RT Master Mix (Takara). The primers used for the RT-qPCR were listed (Supplementary Table 1). The reaction system was prepared according to the manual of TB Green Premix Ex Taq™ (Takara) and conducted on LightCycler96 (Roche). The relative expression of the target genes was normalized to that of elF-4a.

## 3 Results and discussion

### 3.1 Genome assembly and annotation

The genome size of *A. konjac* estimated by the 17-mer depth distribution analysis was 5.67 Gb (Supplementary Fig. 1, Supplementary Table 2), which was somewhat smaller than estimates by previous flow cytometry analysis (approximately 6.33 Gb) [19]. And the heterozygosity estimated was around 0.96% (Supplementary Table 2). The genome of *A. konjac* was sequenced with a combination of Illumina short-read (118×), PacBio (101×) and 10X Genomics (83×) libraries (Supplementary Table 3). The final genome assembly of *A. konjac* was 5.60 Gb with a contig N50 of 1.20 Mb (Table 1). A total of 90.38% of the original assembly (5.06 Gb) were anchored into 13 pseudo-chromosomes by Hi-C (Supplementary Fig. 2). There were a total of 4747 gaps in the genome assembly, and the number of gaps per chromosome ranged from 207 to 584 (Table 2).

**Table 1:** Statistics for the genome assembly of *Amorphophallus konjac*

	**Length**		**Number**	
	Contig* (bp)	**Scaffold (bp)**	Contig*	**Scaffold**
Total	5,598,080,859	5,598,555,559	8,425	3,678
Max	14,753,098	612,111,917	–	–
N50	1,197,616	419,331,422	1,354	6
N90	341,942	212,178,970	4,644	13

Note: *, Contig after scaffolding; N50 and N90 refer to the size above which 50% and 90% of the total length of the sequence assembly can be found.

**Table 2:** Length and gap numbers in each chromosome of the genome assembly

Chromosome ID	Length (Mb)	Number of gaps
Chr_1	273.68	354
Chr_2	486.84	353
Chr_3	360.78	316
Chr_4	444.10	335
Chr_5	612.11	536
Chr_6	483.80	584
Chr_7	419.33	400
Chr_8	351.21	305
Chr_9	365.51	307
Chr_10	463.54	455
Chr_11	313.90	305
Chr_12	273.00	290
Chr_13	212.18	207
Total	5598.56	4747

Alignments of Illumina short reads against the genome assembly revealed a mapping rate of 99.16%, covering 99.75% of the assembled genome (Supplementary Table 4). When aligning reads of RNA-Seq datasets generated from different tissues (leaf, flower, root and corm) against the assembly, an average mapping rate of 93.22% was achieved (Supplementary Table 5). BUSCO analysis found 1343 (83.2%) complete gene models and 69 (4.3%) fragmented gene models out of 1614 genes (Supplementary Table 6). The BUSCO result was similar to those in closely related species, e.g. *C. esculenta* (85.7%) [20] and *S. polyrhiza* (86%) [41]. The CEGMA revealed that 95.16% of the 248 core protein-coding genes were recovered in the genome assembly (Supplementary Table 7). We further calculated the LAI score for genomes of *A. konjac*, *C. esculenta* and *S. polyrhiza*, which were 14.43, 14.49 and 12.24, respectively (Supplementary Table 8). Together, these results show that our genome assembly of *A. konjac* was of high quality.

Protein-coding genes were predicted by combining *de novo*, homolog-based search, and transcriptome methods, resulting in a total of 44,333 genes (Fig. 1E, Supplementary Table 9, Supplementary Fig. 3). The average transcript length was 11,382.9 bp, the coding sequence length was 1094.1 bp, and exon number per gene was 4.21 (Supplementary Table 10, Supplementary Fig. 4). Among the 44,333 genes, 91.5% (40,561) could be functionally annotated against public databases (Supplementary Table 11, Supplementary Fig. 5). Annotation of noncoding RNA genes yielded 1202 miRNAs, 725 tRNAs, 1640 rRNAs and 4696 snRNAs (Supplementary Table 12). Repetitive sequences were analyzed by combining *de novo* prediction and a homology-based search, resulting in a final prediction of 80.6 % of the genome consisting of repetitive sequences (Supplementary Tables 13 and 14).

### 3.2 Genome evolution of *A. konjac*

**Fig. 2:**
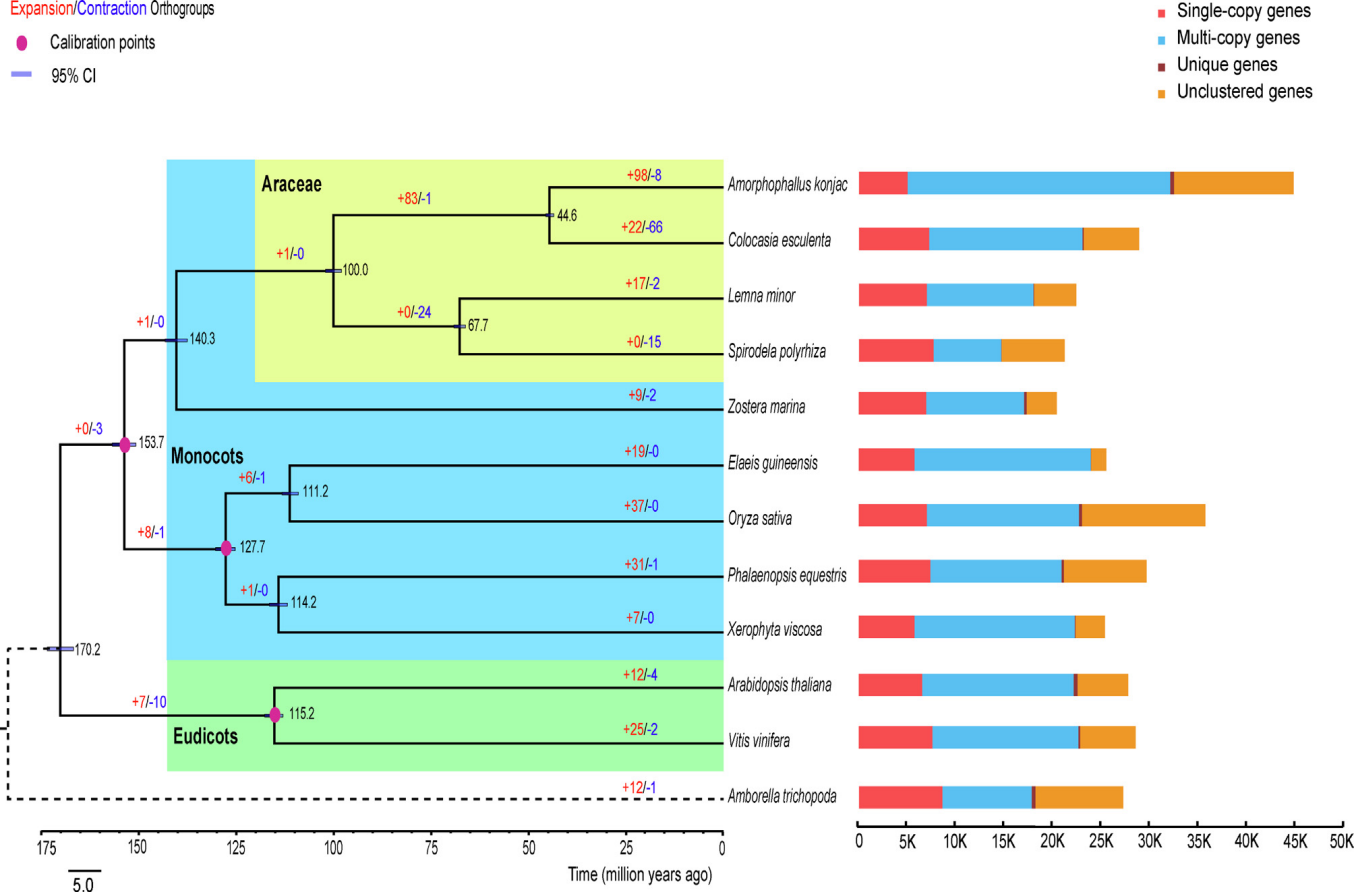
Phylogenetic tree of 12 plant species and evolution of gene families. Left, the phylogeny of 12 species. Black numerical value beside each node shows the estimated divergence time (million years ago), and red circle indicates the node age calibration point. Right, the distribution of single-copy, multiple-copy, unique and unclustered genes for each species.

A total of 12,304 gene families comprising 32,057 genes were identified in *A. konjac* genome (Supplementary Table 15). Compared to *A. thaliana*, *O. sativa* and two Araceae plants (*C. esculenta* and *L. minor*), 561 families are specific in *A. konjac* and 11,743 families are shared with other plants (Supplementary Fig. 6). To infer the phylogenetic position of *A. konjac*, we used 397 single-copy genes from the genomes of 12 species to construct a phylogenetic tree. The ML tree supported a monophyletic clade composed of *A. konjac* and *C. esculenta*, the estimated divergence time of which was approximately 44.6 million years ago (Fig. 2). This finding was in accordant with previous results that Lemnoideae was phylogenetically distinctive [42]. The deducted divergence time of Araceae in this study was about 140 Mya, which was consistent with previous studies (around 138 Ma) [43]. The phylogeny also suggested a relatively close relationship between *A. konjac*, *C. esculenta*, *L. minor* and *S. polyrhiza*. The above four species, along with *Z. marina*, belong to the Alismatales, which evolved as a sister clade to other major monocots (Arecales, Poales, Asparagales and Pandanales) (Fig. 2). In addition, we found that 98 gene families were expanded in *A. konjac*, while 8 families experienced losses (Fig. 2). The expanded genes in *A. konjac* were enriched for gene ontology (GO) terms like ‘binding’, ‘catalytic activity’, ‘metabolic processes’, and ‘cellular process’ (Supplementary Fig. 7).

**Fig. 3:**
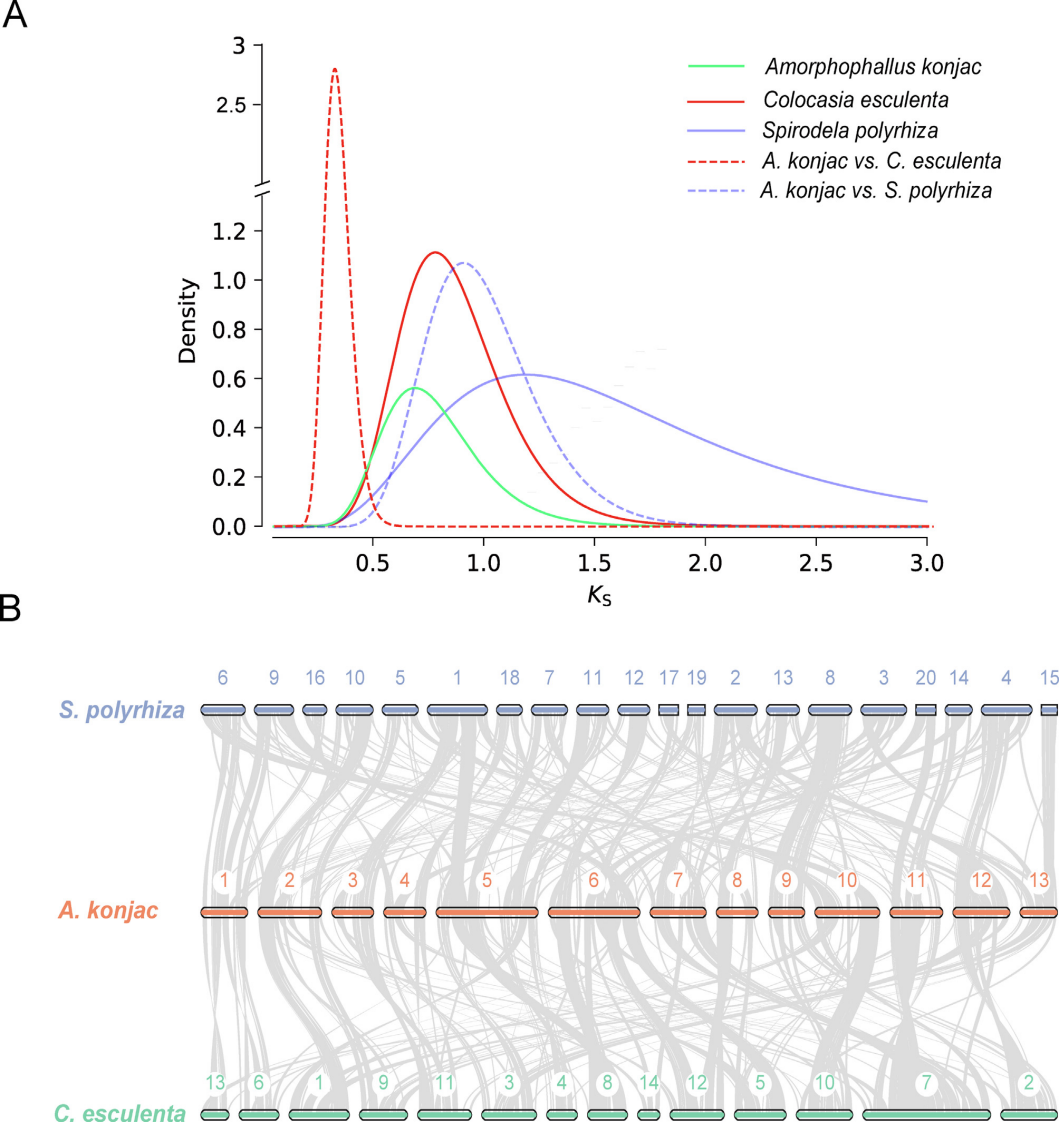
Distribution of synonymous substitution levels (*K*s) of syntenic orthologous (A) and collinearity patterns between paralogous genes of *S. polyrhiza*, *A. konjac* and *C. esculenta* (B).

**Fig. 4:**
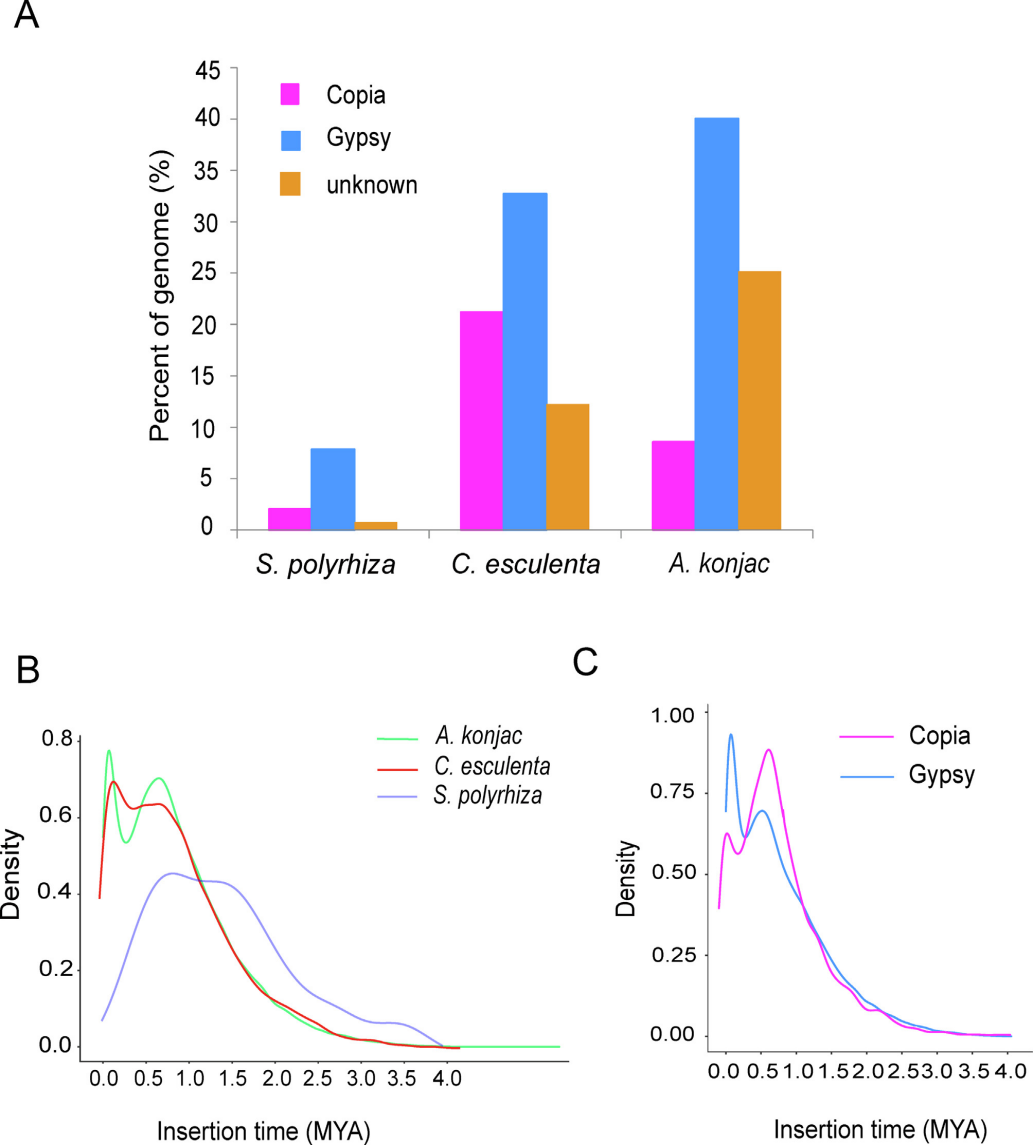
LTR analysis for genomes of *S. polyrhiza*, *A. konjac* and *C. esculenta*. (A) The LTR content in genomes of *S. polyrhiza*, *A. konjac* and *C. esculenta*. (B) The estimated insertion times of LTR in genomes of the three species. (C) Distribution of insertion times of Gypsy and Copia retrotransposons in *A. konjac* genome.

Genome expansion in plants is primarily driven by whole-genome duplication (WGD) events and the proliferation of transposable elements (TEs) [21]. Distributions of the synonymous substitution rates (*K*s) for paralogs of *A. konjac* showed a peak at approximately 0.8, and similar peaks were also identified around *K*s value of 1.0 in *C. esculenta* and *S. polyrhiza* (Fig. 3A, Supplementary Fig. 8). Previous studies reported that *S. polyrhiza* and *C. esculenta* had undergone two separated but time-close WGD events [20], [44]. The *K*s distributions and the genomic collinearity patterns among *A. konjac*, *S. polyrhiza* and *C. esculenta* suggested that *A. konjac* shared both WGD events with these two species (Fig. 3A and 3B). We also identified an abundance of repetitive sequences (about 4.51 Gb), which constituted 80.6 % of the genome assembly (Supplementary Tables 13 and 14). This percentage was much higher than that of *S. polyrhiza* (13.06 %) [44]. Long terminal repeat (LTR) retrotransposons accounted for 74.04 % of the *A. konjac* genome (Fig. 4A, Supplementary Table 14). In comparison with *S. polyrhiza*, LTRs in genomes of *A. konjac* and *C. esculenta* showed two recent bursts approximately 0.1 MYA and 0.6–0.7 MYA, respectively (Fig. 4B). The recent expansion of transposable elements in *A. konjac* may explain most of the 35-fold difference in genome size between *A. konjac* and *S. polyrhiza*. Although both Gypsy and Copia went through two LTR burst events in *A. konjac*, the increased proportion during two events differed between two superfamilies (Fig. 4B and 4C, Supplementary Table 8).

### 3.3 Biosynthesis of konjac glucomannan

**Fig. 5:**
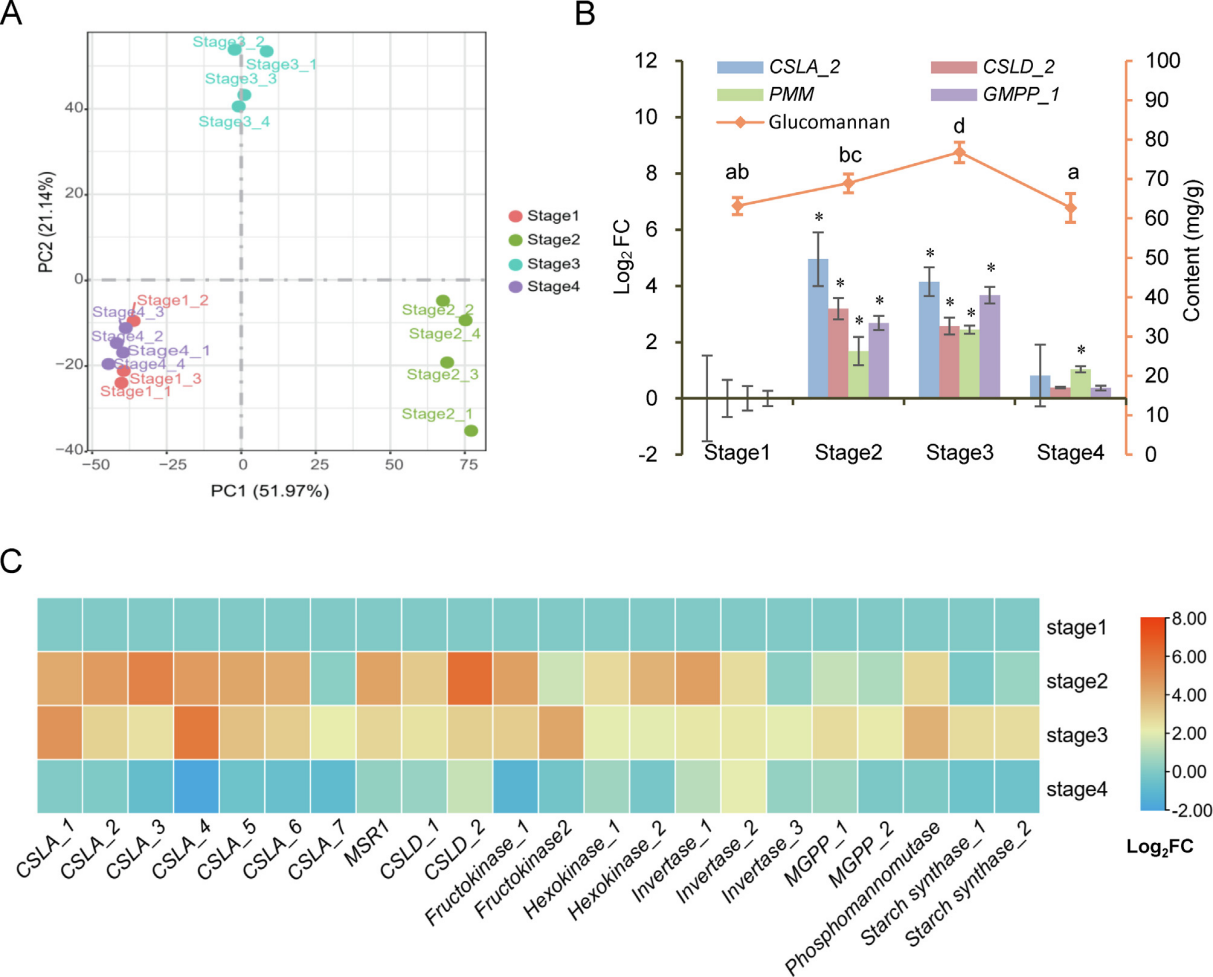
Transcriptome and RT-qPCR analyses for KGM biosynthesis. (A) Principal component analysis (PCA) of 15 *A. konjac* corm samples. (B) RT-qPCR and measurement of KGM content. Values represent means ± SD. Asterisks indicate statistical significance using student’s *t*-test (*P* < 0.05, n = 3) and one-way ANOVA with post hoc Tukey HSD test is applied to compare KGM content of four stages (*P* < 0.01, n = 4). (C) Heatmap of KGM biosynthesis-related genes that are highly expressed in stage 2 and/or stage 3. The threshold is Log_2_ FC (stageN/stage1) > 2 (N = 2 or 3, *P* < 0.05).

RNA-seq was performed for developmental corms collected from four stages of the vegetative growth circle (Supplementary Tables 5 and 16). The heatmap displays a high positive correlation between the biological repeats of each stage (Supplementary Fig. 9). Similarly, principal component analysis (PCA) shows that the biological repeats of stage 2 and stage 3 are clustered into distinct groups, whereas samples from both stage 1 and stage 4 form a separate group (Fig. 5A). This is further supported by the observation that more differentially expressed genes (DEGs) are found in stage 2 and stage 3 compared with stage 4 when using stage 1 as a control (Supplementary Fig. 10). GO enrichment analysis on DEGs revealed that the top over-represented biological processes are associated with cellular carbohydrate metabolism, including glucan metabolic process, in both stage 2 and stage 3 (Supplementary Table 17), indicating the high carbohydrate metabolism activity during this period. As expected, glucomannan content measurement showed a substantial increase from stage 2 to stage 3 (Fig. 5B).

Based on previous studies on glucomannan biosynthesis [7], [10], [12], 97 putative genes involved in the pathway and their expression pattern were identified (Supplementary Table 18). Particularly, one of these coded proteins is called MSR1, which is a homolog of AtMSR1 and firstly identified using BLASTP against our *A*. *konjac* protein database (Supplementary Table 19). Based on pairwise sequence alignment, AkMSR1 shows 55.2 % sequence identity and 72.0 % sequence similarity to AtMSR1. To identify the key genes in glucomannan biosynthesis, genes that are highly expressed in stage 2 and/or stage 3 were extracted (Fig. 5C and Supplementary Table 20). And six of these genes could be also identified by Pearson correlation analysis of gene expression and glucomannan content (r > 0.95, *p*-value < 0.05) (Supplementary Table 21). In addition, RT-qPCR was also applied for four genes (cellulose synthase-like A (*CSLA*_*1*), Cellulose synthase-like D (*CSLD*_*2*), phosphomannomutase (*PMM*) and GDP-mannose pyrophosphorylase (*GMPP*_*1*)), which displays a similar expression pattern consistent with the RNA-seq data (Fig. 5B and Supplementary Table 22).

**Fig. 6:**
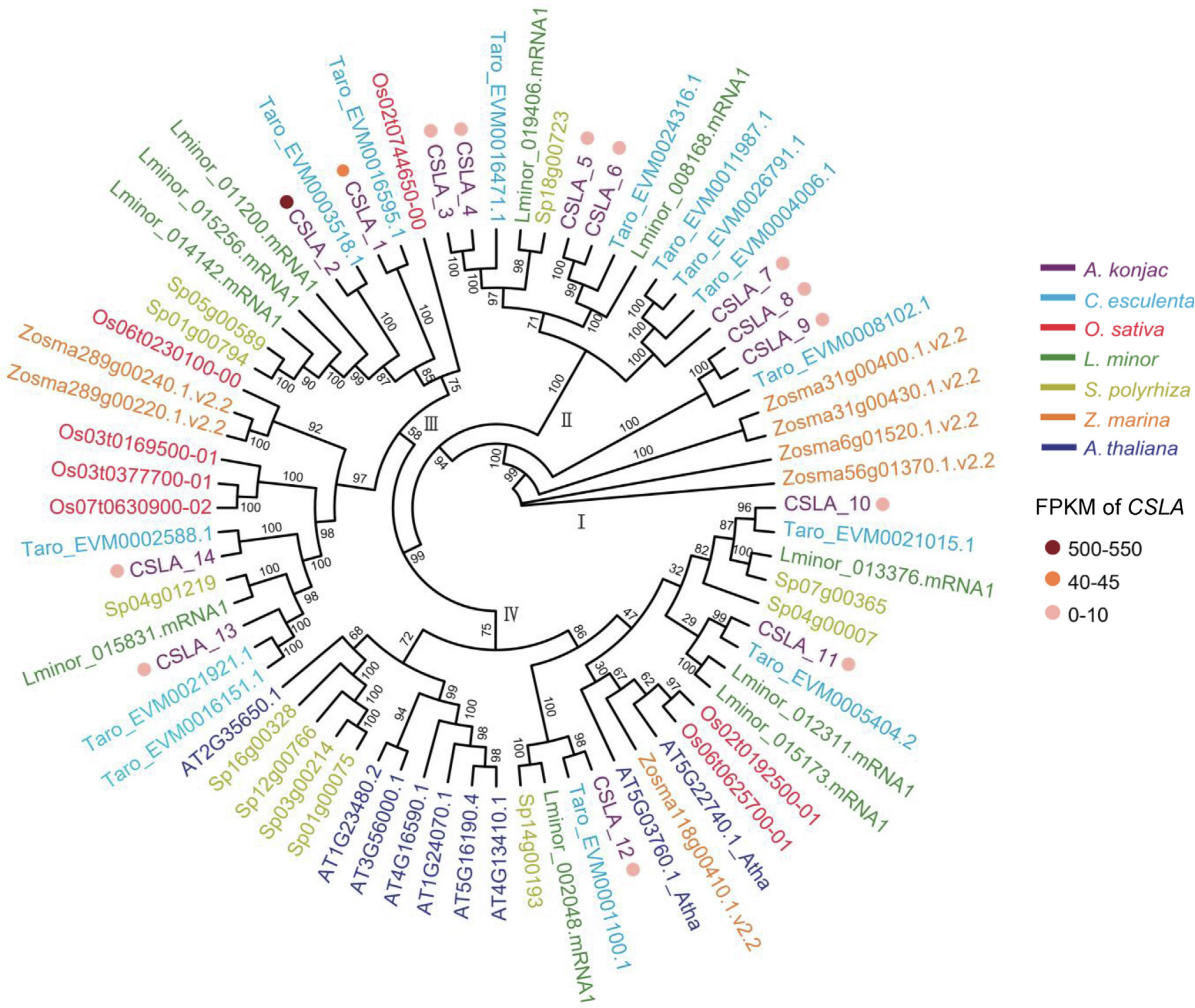
Maximum likelihood (ML) tree of CSLA family of enzymes. Different colors represented different species, and only the FPKM values of *CSLA* genes at stage 2 were shown by colored circles.

**Fig. 7:**
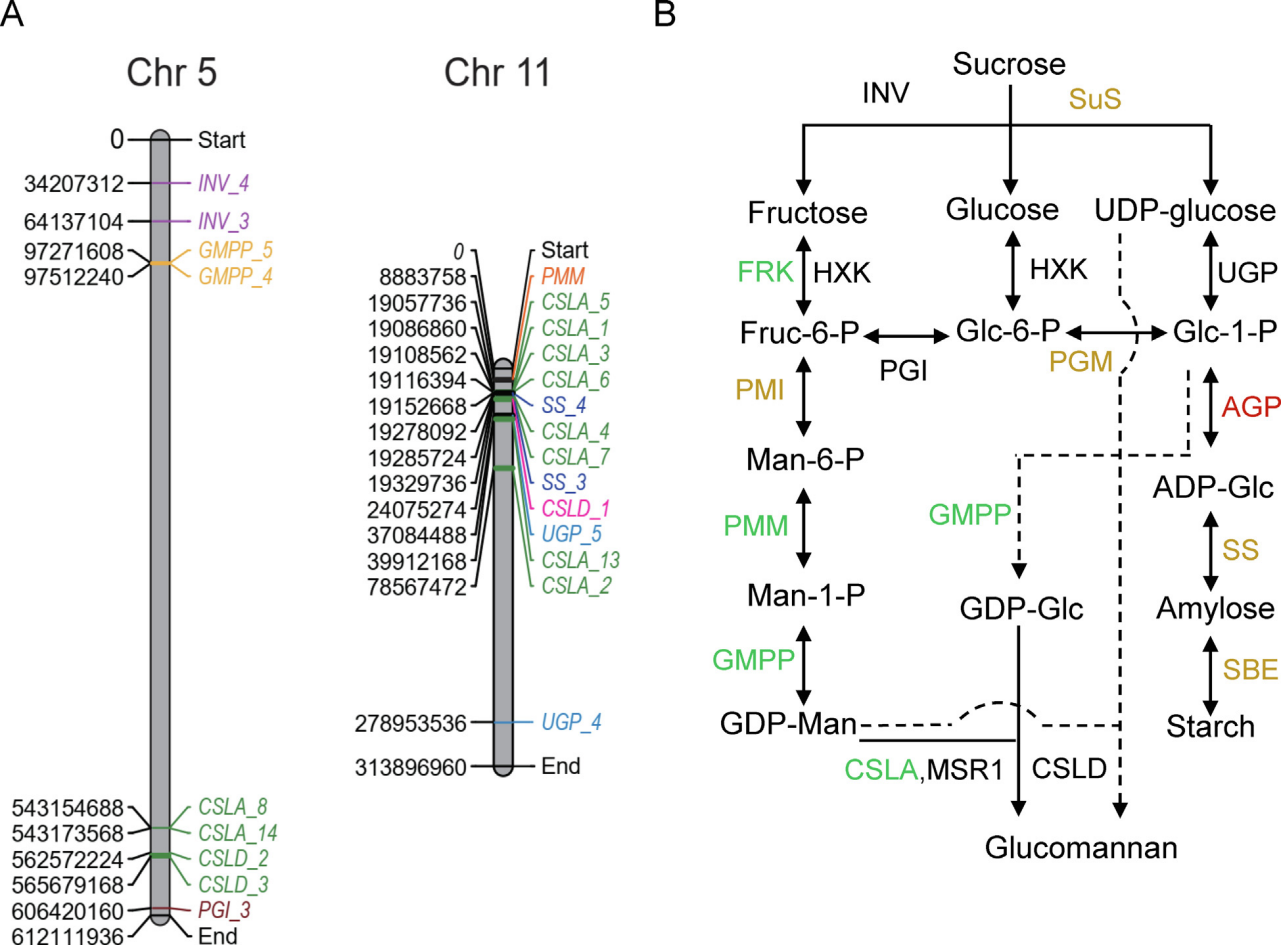
Chromosome positions of KGM synthesis-related genes and putative biosynthetic pathway of KGM. (A) Positions of KGM synthesis-related genes distributed on chromosomes 5 and 11. (B) Putative biosynthetic pathway of KGM. Group 1, group 2 and group 3 are highlighted in green, orange and red, respectively. Dash lines represent speculative pathways. Sucrose synthase (SuS), invertase (INV), phosphoglucose isomerase (PGI), phosphoglucomutase (PGM), phosphomannose isomerase (PMI), phosphomannomutase (PMM), starch synthase (SS), GDP-mannose pyrophosphorylase (GMPP), UDP-glucose pyrophosphorylase (UGP), ADP-glucose pyrophosphorylase (AGP), fructokinase (FRK), hexokinase (HXK), starch branching enzyme (SBE), cellulose synthase-like A (CSLA), Cellulose synthase-like D (CSLD), mannan-synthesis related 1 (MSR1).

The phylogenetic tree clustered CSLA genes into four subgroups (Fig. 6). Like other multiple-copy pathway genes in KGM biosynthesis, the expression levels of CSLA family members vary significantly (Supplementary Table 18). In particular, the phylogenetically-close *CSLA_1* and *CSLA_2* were highly detected in stage 2, which are supposed to play a major role in KGM biosynthesis (Fig. 6). Previous studies of recombinant CSLA proteins have demonstrated that a single CSLA protein in a heterologous host is sufficient for glucomannan synthesis using mannose and glucose [7], [45]. It is possible that all CSLA proteins are involved in mannan synthesis, while only certain proteins may catalyze the synthesis of other polysaccharides, such as KGM [46]. In addition, our phylogenetic analysis indicated that all CSLA of *A. thaliana* were clustered into group Ⅳ, while CSLA_1 and CSLA_2 were found in group III. Some researchers have suggested that a clade of CSLA proteins presented only in monocots may have divergent functions [47], [48]. Besides, the variation in gene expression was also observed in CSLD family (Supplementary Fig. 11). Functional characterization of these genes in the future will strengthen the information on the biosynthesis of KGM. We also visualized the location of all KGM synthesis-related genes on chromosomes (Supplementary Fig. 12). Interestingly, tandem gene duplication was observed for eight out of 14 *CSLA* members on chromosomes 5 and 11 (Fig. 7A), which may have a positive effect on KGM biosynthesis.

In addition to changes in gene expression pattern, transcript abundances of KGM biosynthesis-related genes were also analyzed. According to total FPKM (fragments per kilobase per million), the top 11 highly expressed genes were divided into three groups. Group Ⅰ contains genes that are highly expressed in stage 2 and 3 including *CSLA*, *GMPP*, *PMM* and fructokinase (*FRK*) (Supplementary Table 23). Group Ⅱ contains genes that are highly expressed at later stages (stage 3 or 4) including starch synthase (*SS*), starch branching enzyme (*SBE*), phosphoglucomutase (*PGM*), phosphomannose isomerase (*PMI*) and sucrose synthase (*SuS*) (Supplementary Table 23). Only one gene called ADP-glucose pyrophosphorylase (*AGP*) belongs to group III and seems to be constitutively expressed (Supplementary Table 23). Combined with the gene expression data, the proposed pathway strongly suggests that *A. konjac* synthesizes KGM mainly at a middle stage but switches to the biosynthesis of starch at a later stage during corm development (Fig. 7B). To sum up, temporal regulation of gene expression, such as *CSLA*, *MSR1*, *GMPP*, *PMM* and *FRK*, may play a key role in KGM biosynthesis.

## 4 Conclusions

As one of the largest and most diverse flowering plant families, Araceae contains a number of species that are important sources of food (e.g. *C. esculenta*, *Cyrtosperma merkusii*, *A. paeoniifolius*, *A. konjac*), medicine, fiber and ornament [18]. Given the great economic importance, the high-quality genome assembly presented here will provide valuable genomic resources for gene function study and future breeding of *A. konjac*. Despite its large genome size, we failed to reveal additional WGD events except for two for the total family. Instead, the recent expansion of transposable elements likely leads to the large genome size of this species. Besides, several key genes involved in KGM biosynthesis were identified based on genomic and transcriptomic data. Temporal regulation of gene expression, such as *CSLA*, *MSR1*, *CSLD*, *GMPP* and *PMM*, appears to play a key role in KGM biosynthesis. Future studies need to answer how temporal regulation of the key genes is achieved as well as the exact role of MSR1 in KGM synthesis. Overall, these key genes provide potential candidates for molecular breeding of *A. konjac*. The improvement of yield and the glucomannan content through genetic engineering approaches is in prospect.

## Declaration of Competing Interest

The authors declare that they have no known competing financial interests or personal relationships that could have appeared to influence the work reported in this paper.
