# Supplementary material for: A chromosome-level genome assembly of Amorphophallus konjac provides insights into konjac glucomannan biosynthesis
Source: Comput Struct Biotechnol J. 2022 Feb 15;20:1002–11. doi: 10.1016/j.csbj.2022.02.009 (PMC8860920; doi:10.1016/j.csbj.2022.02.009)
Supplement: Supplementary data 1 [file mmc1.docx]

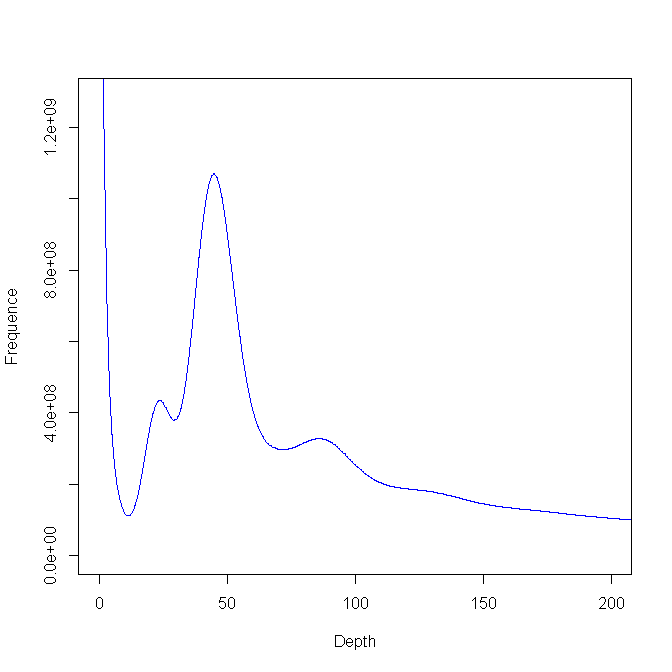


**Supplementary Figure 1** Distribution of 17-mer frequency in the genome of *Amorphophallus konjac*.


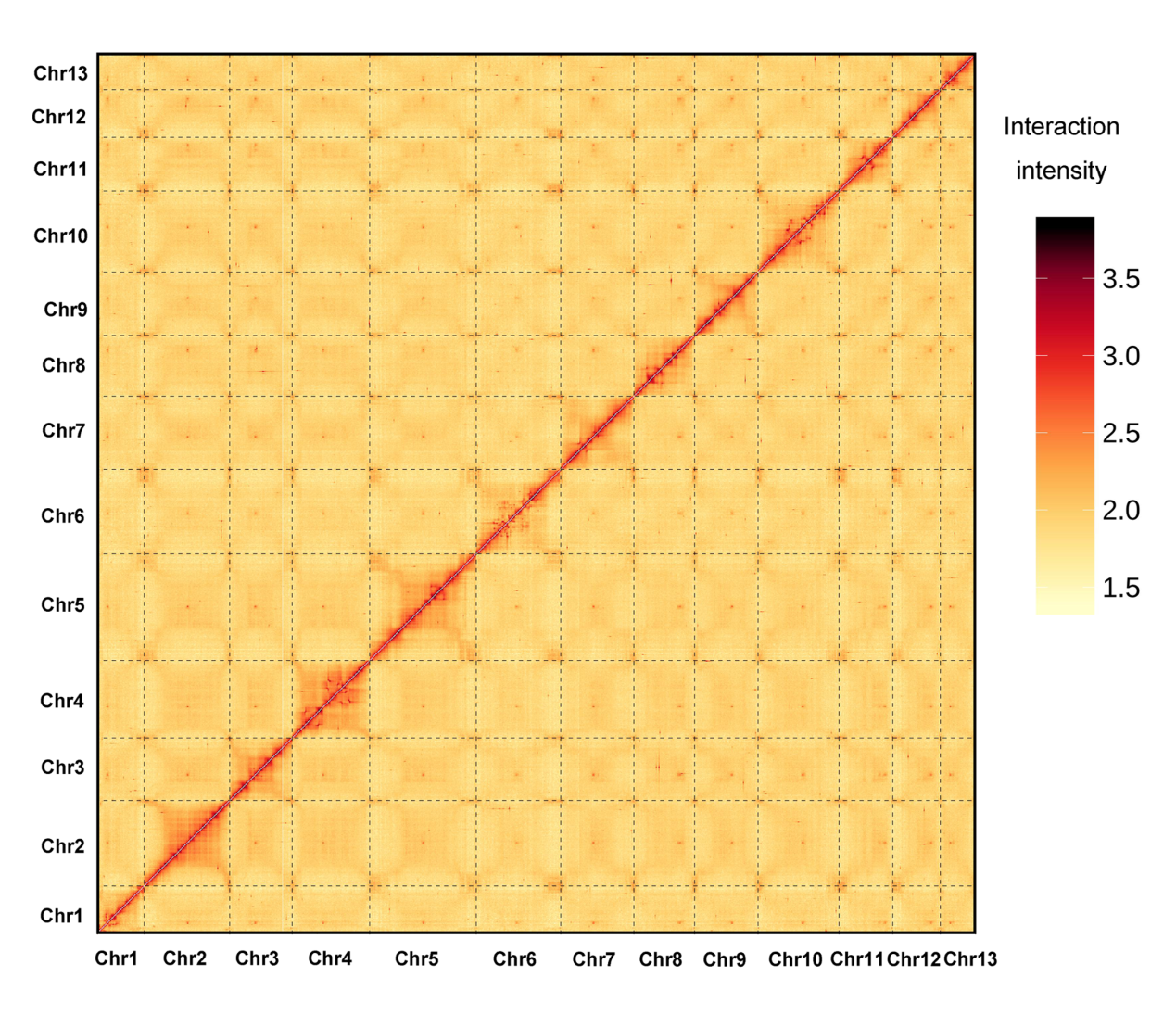


**Supplementary Figure 2** Heatmap showing Hi-C interactions at 500 kb resolution, with intrachromosomal interactions displayed as anti-diagonal patterns.


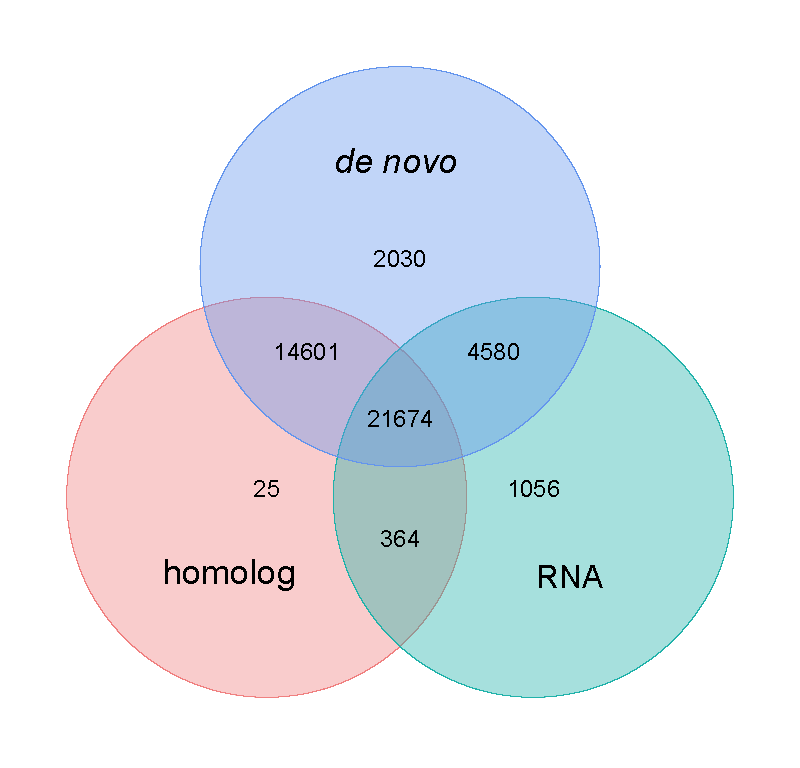


**Supplementary Figure 3** Venn diagram showing the predicted genes in *de novo*, homolog-based and RNA predicted methods.


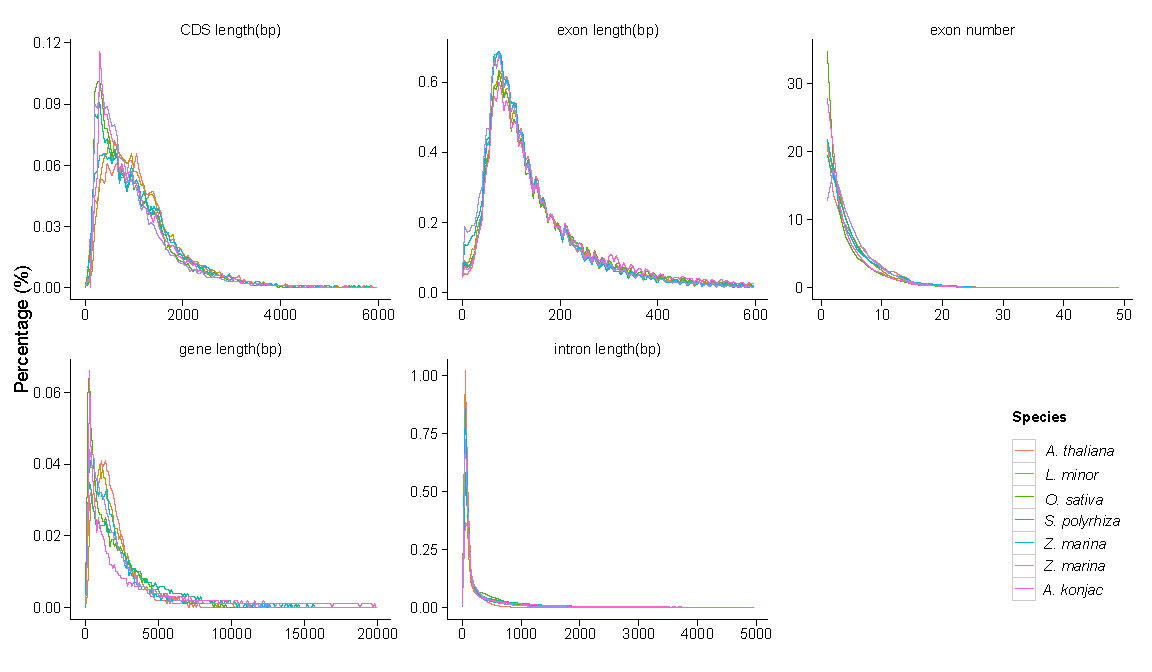


**Supplementary Figure 4** Comparison of gene structure characteristics in *A. konjac* to that in other plants. a, CDS length; b, exon length; c, exon number; d, gene length; e, intron length.


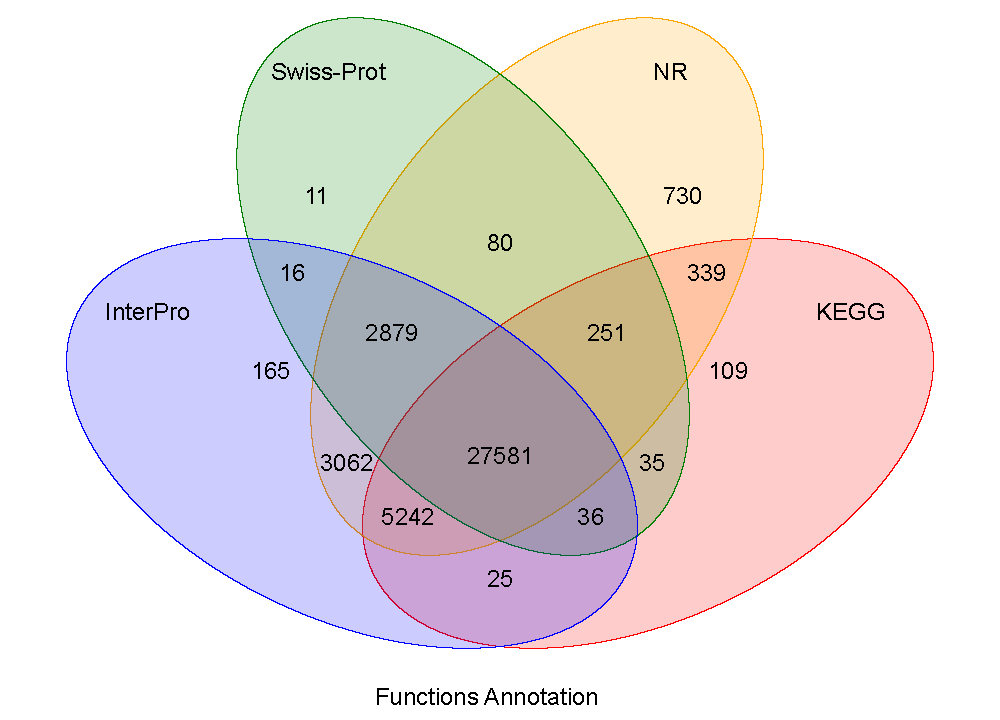


**Supplementary Figure 5** Venn diagram showing the function annotations by different databases.


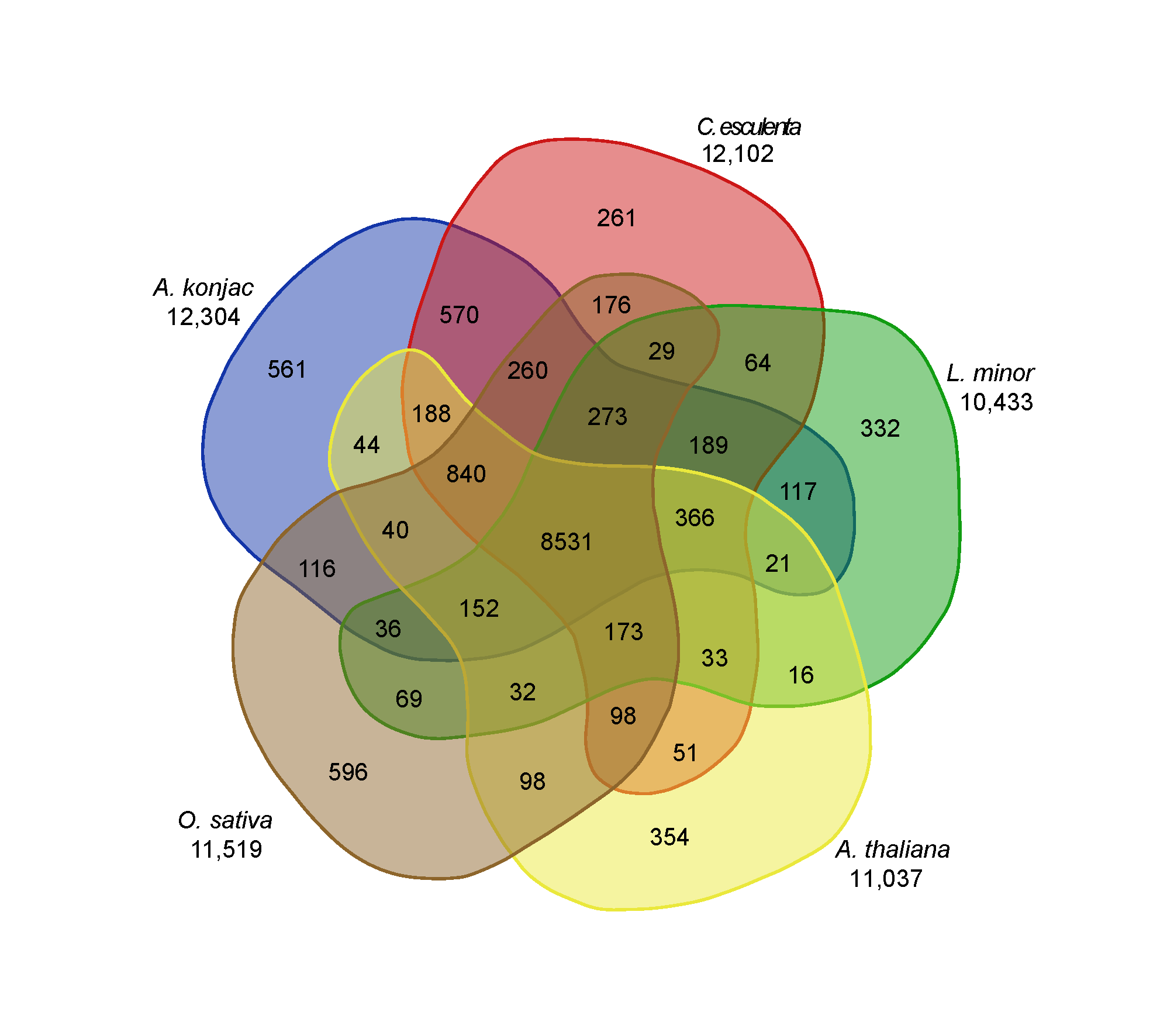


**Supplementary Figure 6** Venn diagram showing orthologous groups shared between *A. konjac* and four other plants. Each number represents a gene family number.


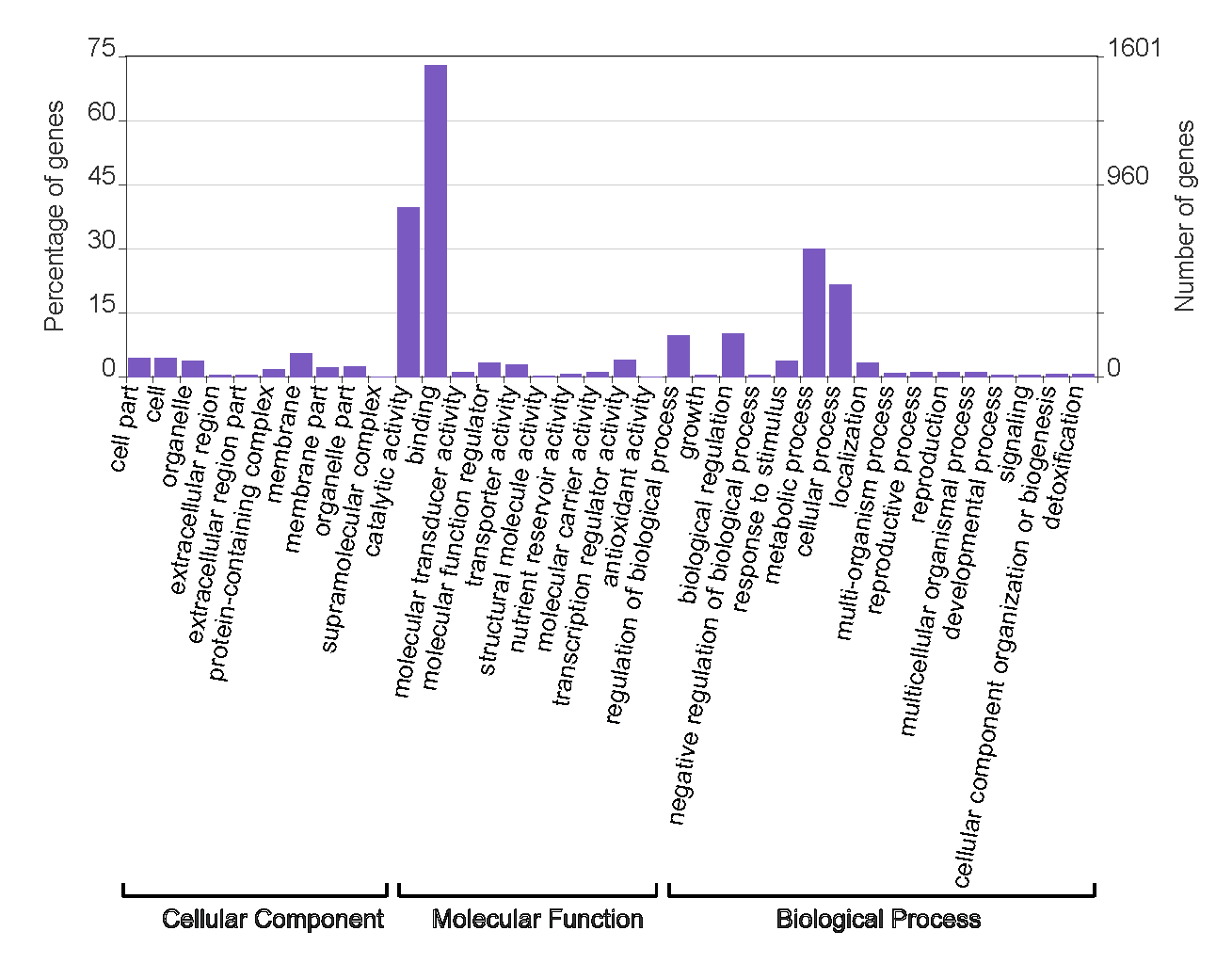


**Supplementary Figure 7** Diagram showing the gene ontology (GO) categories of expanded genes in *A. konjac*.


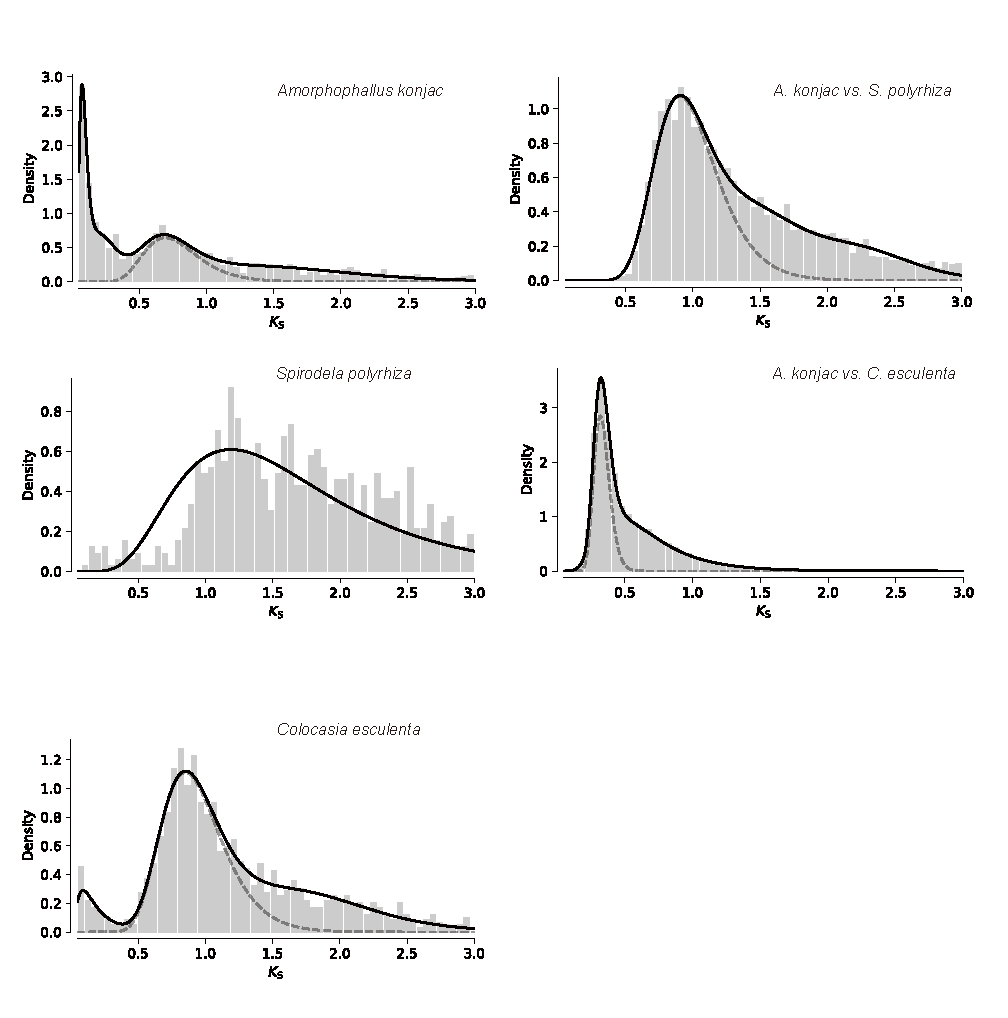


**Supplementary Figure 8** *K*s distribution for paralogs in *A. konjac*, *S. polyrhiza* and *C. esculenta*, and for orthologs between them. Dashed lines represent individual WGDs are fitted by a mixture model (BGMM).


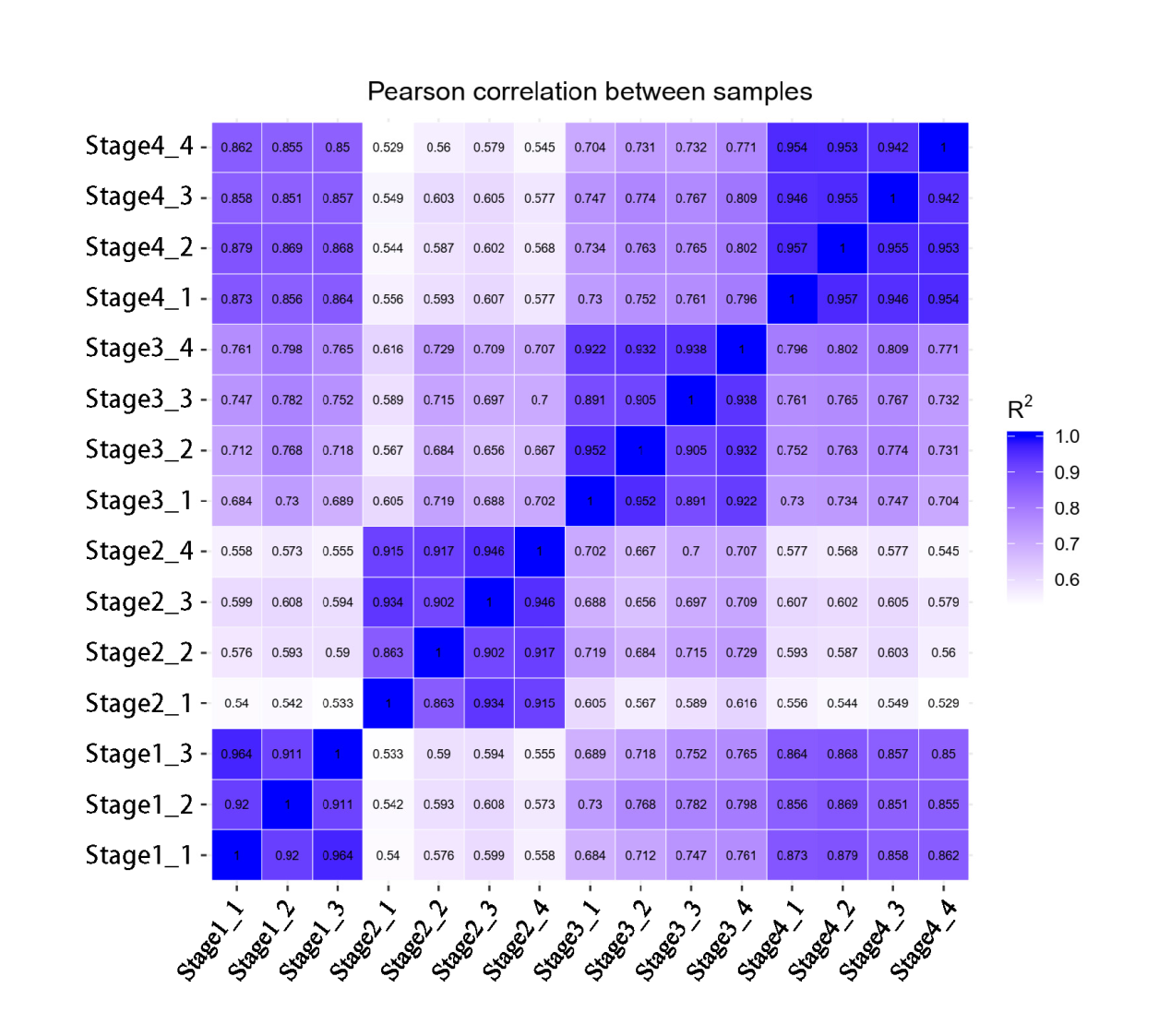


**Supplementary Figure 9** The heatmap of positive correlation between the biological repeats of each stage.


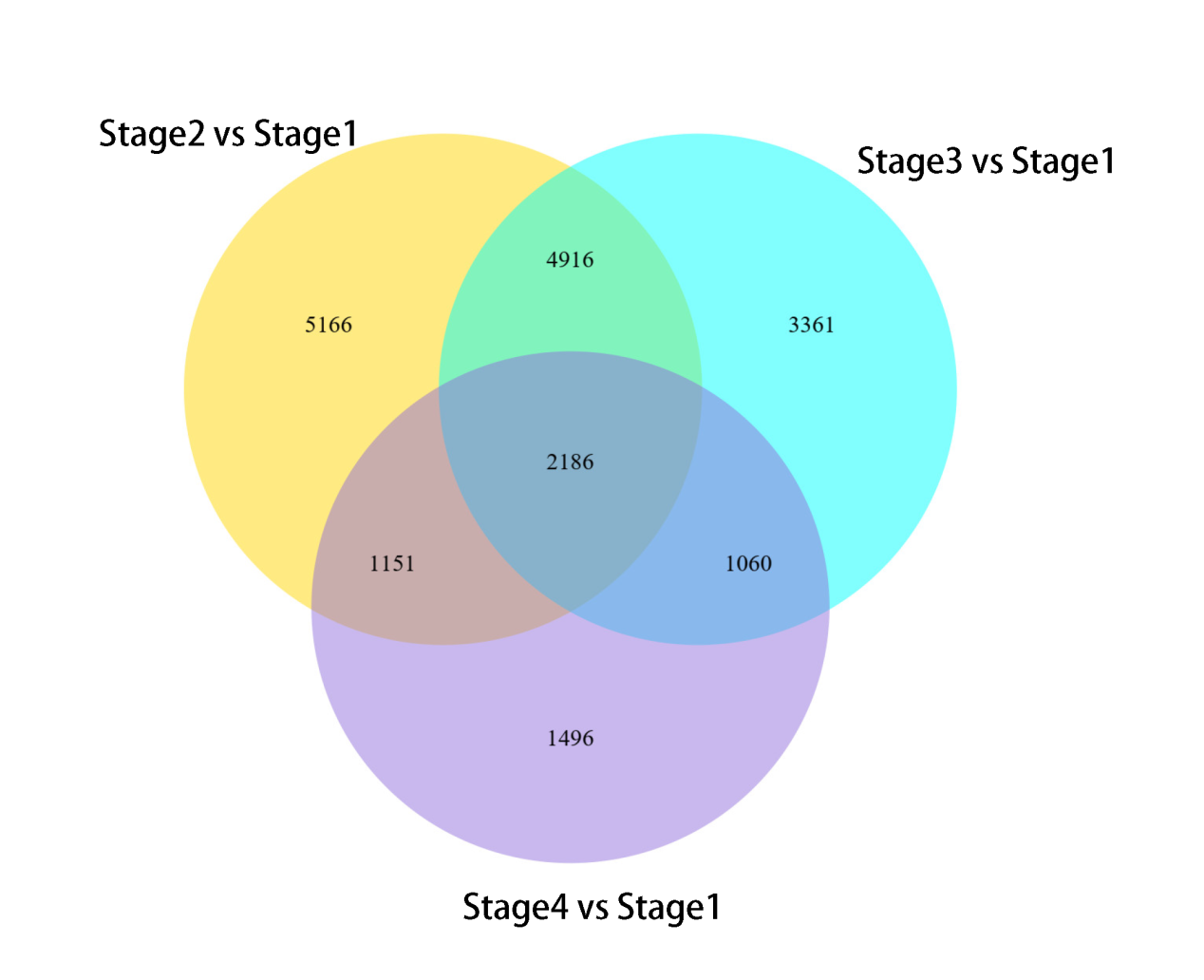


**Supplementary Figure 10** Venn diagram showing the differentially expressed genes when comparing stage 2, stage 3 and stage 4 to stage 1.


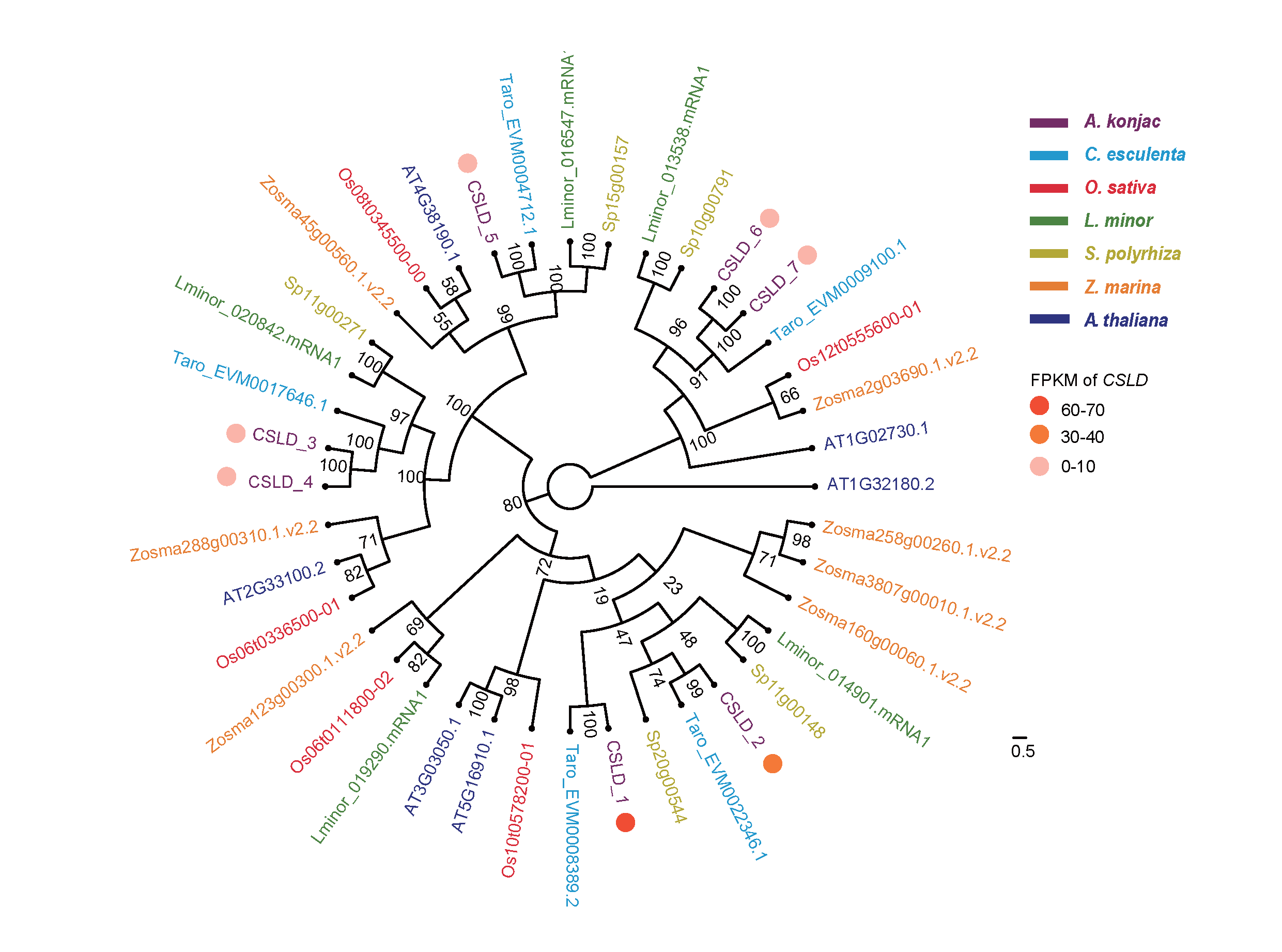


**Supplementary Figure 11** Maximum likelihood (ML) tree of CSLD family of enzymes. Different colors represented different species, and the FPKM values of CSLD genes at stage 2 were shown by colored circles.


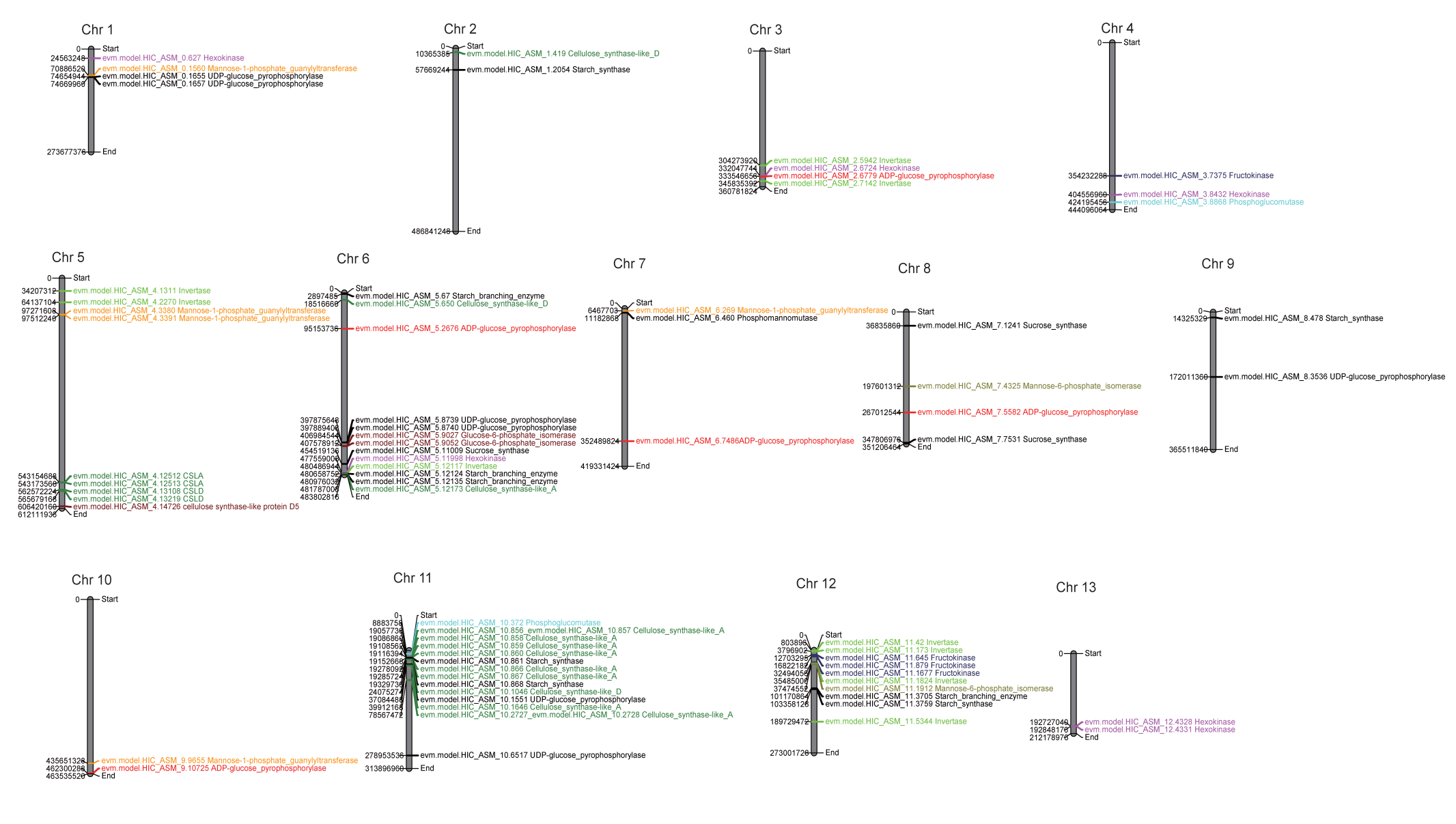


**Supplementary Figure 12** Positions of KGM synthesis-related genes distributed on 13 chromosomes.
